# Understanding, treating, and renaming grandiose delusions: A qualitative study

**DOI:** 10.1111/papt.12260

**Published:** 2019-11-29

**Authors:** Louise Isham, Laura Griffith, Anne‐Marie Boylan, Alice Hicks, Natalie Wilson, Rory Byrne, Bryony Sheaves, Richard P. Bentall, Daniel Freeman

**Affiliations:** ^1^ Oxford Cognitive Approaches to Psychosis (O‐CAP) Department of Psychiatry University of Oxford UK; ^2^ Oxford Health NHS Foundation Trust UK; ^3^ Health Services Management Centre University of Birmingham UK; ^4^ Nuffield Department of Primary Care Health Sciences University of Oxford UK; ^5^ Patient Advisory Group Oxford Cognitive Approaches to Psychosis (O‐CAP) Department of Psychiatry University of Oxford UK; ^6^ The McPin Foundation London UK; ^7^ Psychosis Research Unit Greater Manchester Mental Health NHS Foundation Trust UK; ^8^ Department of Psychology University of Sheffield UK

**Keywords:** delusions of exceptionality, qualitative, harm, maintenance mechanisms, therapy

## Abstract

**Background:**

Grandiose delusions are arguably the most neglected psychotic experience in research.

**Objectives:**

We aimed to discover from patients: whether grandiose delusions have harmful consequences; the psychological mechanisms that maintain them; and what help patients may want from clinical services.

**Design:**

A qualitative interview design was used to explore patients’ experiences of grandiose delusions.

**Method:**

Fifteen patients with past or present experiences of grandiose delusions who were attending psychiatric services were interviewed. Thematic analysis and grounded theory were used to analyse the data.

**Results:**

Participants reported physical, sexual, social, occupational, and emotional harms from grandiose delusions. All patients described the grandiose belief as highly meaningful: it provided a sense of purpose, belonging, or self‐identity, or it made sense of unusual or difficult events. The meaning from the belief was not synonymous with extreme superiority or arrogance. The meaning obtained appeared to be a key driver of the persistence of the beliefs. Other maintenance factors were subjectively anomalous experiences (e.g., voices), symptoms of mania, fantasy elaboration, reasoning biases, and immersive behaviours. Participants described insufficient opportunities to talk about their grandiose beliefs and related experiences and were generally positive about the possibility of a psychological therapy.

**Conclusions:**

We conclude that grandiosity is a psychologically rich experience, with a number of maintenance factors that may be amenable to a targeted psychological intervention. Importantly, the term ‘grandiose delusion’ is an imprecise description of the experience; we suggest ‘delusions of exceptionality’ may be a credible alternative.

**Practitioner points:**

- Harm from grandiose delusions can occur across multiple domains (including physical, sexual, social, occupational, and emotional) and practitioners should assess accordingly.
- However, grandiose delusions are experienced by patients as highly meaningful: they provide a sense of purpose, belonging, or self‐identity, or make sense of unusual or difficult events.
- Possible psychological maintenance mechanisms that could be a target for intervention include the meaning of the belief, anomalous experiences, mania, fantasy elaboration, reasoning biases, and immersive behaviours.
- Patients are keen to have the opportunity to access talking therapies for this experience. Taking extra time to talk at times of distress, ‘going the extra mile’, and listening carefully can help to facilitate trust.

Grandiose delusions are unfounded beliefs that one has special powers, wealth, mission, or identity (Leff, Fischer, & Bertelsen, 1976). Despite being a common type of delusion (Appelbaum, Robbins, & Roth, 1999; Goodwin & Jamison, 2007) – occurring in about half of patients diagnosed with schizophrenia and two thirds of patients with bipolar disorder(Knowles, McCarthy‐Jones, & Rowse, 2011) – they have been remarkably neglected as a specific focus of research and clinical practice. Indeed, although theoretical discussions about grandiose beliefs date back more than 100 years (Bleuler, 1950; Freud, 1911), very little in the way of empirical research has been conducted (Knowles *et al.*, 2011), and only a handful of studies test hypotheses regarding causal or maintenance mechanisms. This dearth of research activity is particularly apparent when compared to the extensive literature focusing on other psychotic experiences such as persecutory delusions and auditory hallucinations.

This apparent disparity may have arisen for several reasons. There may be a perception that grandiose delusions represent a more benign presentation in non‐affective psychosis and that they will not be distressing or harmful given the focus of the belief. Alternatively, they may be viewed simply as a symptom of mania in affective psychosis, and therefore, it is presumed that research and clinical focus should be on the manic episode rather than the belief *per se*. These assumptions, however, may be erroneous. Both harm and distress can occur with grandiose delusions (e.g., believing one is invincible and stepping into traffic, or believing one is Jesus and will therefore be crucified). Potential maintenance mechanisms (e.g., reasoning biases) beyond mania have been identified (Bortolon, Yazbek, Norton, Capdevielle, & Raffard, 2019; Garety *et al.*, 2012) and others hypothesized (Knowles *et al.*, 2011). Furthermore, factor analytic symptom studies and twin design genetic studies suggest that there are distinct aetiological influences for different psychotic experiences, including grandiosity (Ronald *et al.*, 2014; Zavos *et al.*, 2014), and there is therefore a rationale for the development of experience‐specific models and intervention (Freeman, 2016). Our view is that grandiose delusions require specific research scrutiny.

Our aim was to further understanding directly from patients. Three key areas were examined: the harmful consequences of grandiose delusions, why the beliefs persist, and what patients may want from services. The rationale to intervene is inextricably linked to whether grandiose delusions cause harm, and therefore, we wanted to detail the types of harmful consequences that may occur. If intervention is indicated, then the mechanisms to target to effect change must be known. Preliminary evidence suggests possible roles for reasoning biases, hallucinations, and self‐esteem (Ben‐Zeev, Morris, Swendsen, & Granholm, 2011; Bortolon *et al.*, 2019; Garety *et al.*, 2012) but our understanding of the factors maintaining grandiose delusions is currently very limited. We therefore wanted to generate hypotheses for maintenance factors directly from patient reports. Finally, grandiose delusions increase the risk of a patient being unmotivated to engage in standard treatment (Mulder, Koopmans, & Hengeveld, 2005) but little is known about why this is. Patients may feel that treatments are irrelevant or unhelpful, and such perspectives must be understood in order for an acceptable intervention to be developed. We therefore wanted to learn from patients what they would, and would not, want from clinical services.

## Methods

The study was approved by the NHS Health Research Authority (REC reference: 17/SC/0515).

### Research team

The study was designed and conducted by a team with a range of expertise. This included those with personal experience of grandiose delusions, as well as experts in the development and delivery of psychological models and treatments for psychotic experiences, and in qualitative methodology. This ensured that multiple perspectives were obtained at all stages of the research process, which was invaluable in maximizing the credibility and dependability (or validity and reliability) of the study (Guest, MacQueen, & Namey, 2012a).

### Participants

Participants were sought from clinical teams in Oxford Health NHS Foundation Trust. Inclusion criteria were as follows: aged 16+ years; current/past experience of grandiose delusions held for at least 1 month with at least 50% conviction; and a primary diagnosis of schizophrenia‐spectrum psychosis or bipolar affective disorder. Individuals without capacity to consent, with insufficient comprehension of English, or with primary diagnoses of drug/alcohol/personality disorder, learning disability, or organic syndrome were excluded. Potential participants were identified by their clinical teams and, if consent was given to do so, approached by the lead author who provided information about the study and screened for suitability. The Schedules for Clinical Assessment in Neuropsychiatry (Wing *et al.*, 1990) (items 19.029 delusions of grandiose abilities and 19.030 delusions of grandiose identity) was used to assess grandiose delusions. All participants gave written informed consent.

#### Purposive sampling

Representation across those with (1) current and past grandiose delusions, and (2) affective and non‐affective diagnoses, was prioritized (Richie, Lewis, & Elam, 2014). This was due to anticipated differences in views on harm, treatment, and maintenance factors. Some harms (such as social embarrassment) were anticipated as being potentially more apparent to those with past beliefs, whereas some maintenance factors might be more readily identified in those currently holding a grandiose delusion. Ensuring participants with affective and non‐affective diagnoses were included allowed us to consider these experiences both within and outside of the context of mania. Variation across gender, age, and service experience was also sought where possible.

#### Data saturation

**Figure 1:**
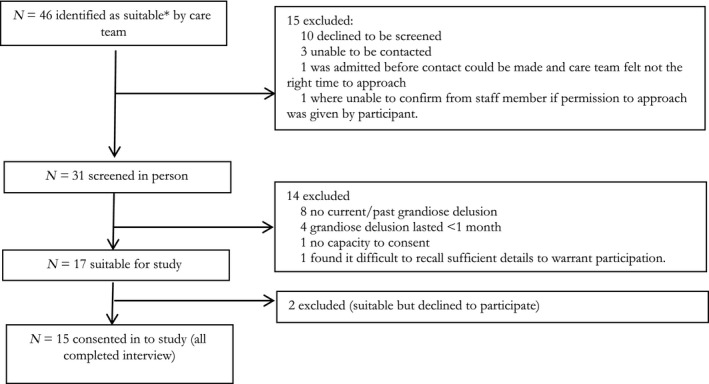
Sample extraction. *NB: A heterogeneous sample was pursued by purposive sampling to include those with current and past grandiose delusions, and affective and non‐affective diagnoses.

Data saturation was considered to have been achieved when no new themes emerged from additional interviews (Fusch & Ness, 2015). In practice, it was felt that this had occurred by the thirteenth participant but a further two participants were recruited to test and confirm this. This resulted in a final sample of 15 participants. Sample extraction details (Figure 1) and participant characteristics (Tables 1 and 2) are provided.

**Table 1:** Sample characteristics (*n* = 15)

Demographic characteristic	Frequency
Age (years)	
16–25	2
26–35	3
36–45	5
46–55	1
56–65	4
Gender	
Male	7
Female	8
Ethnicity	
White British	12
Indian	1
Black British Caribbean	1
Mixed White and Black British	1
Marital status	
Single	8
Engaged	1
Married	3
Divorced	3
Employment	
Employed full time	1
Employed part time	2
Student and part‐time employment	1
Unemployed	11
Diagnoses	
Schizophrenia	4
Schizoaffective disorder	4
Bipolar affective disorder	6
Non‐organic psychotic disorder (working diagnosis due to first presentation)	1
Current/past grandiose belief	
Current belief about current abilities/identity	8
Current belief about past abilities (doesn’t believe currently has abilities)	2
Past belief not currently subscribed to	5
Service context at the time of interview	
Community mental health team (CMHT)	11^2^
Early intervention in psychosis (EIP) team	2
Acute psychiatric inpatient setting	2
Experience of psychiatric admission	
At least one psychiatric admission	13
No	2
Self‐reported experience of any talking therapy (not necessarily for grandiose belief)	
No	4
Yes (incl. GP counselling, individual psychotherapy or counselling (private), individual CBT (NHS), mindfulness group therapy (NHS), ward‐based psychosis group (NHS))	11

Note

Demographic details were provided by participants except for diagnosis and service context (identified at point of referral).

^a^ One participant was open to CMHT at the time of interview but had been discharged from hospital the previous day.

**Table 2:** Content of grandiose beliefs discussed during interview

Pseudonym	Marital status	Employment	Diagnosis	Current service	Past or current belief	Belief(s)
Bob	Single	Student and Employed‐PT	BD	EIP	Past	I have the capacity to become the next Messiah and am on a special pathway towards achieving this.
Mark	Divorced	Unemployed	SzA	AMHT^4^	Current	I am working undercover for the security services.
Mandy	Married	Unemployed	SzA	AMHT	Current	I am a Goddess and the daughter of God with whom I have a special relationship. In the next world, I will be married to Jesus, will have special powers to help people, and will bring peace to the world.
Kit	Single	Employed‐PT	BD	Inpatient	Current	I am Jesus. I am the one son of God. I have special spiritual and mystical abilities which allow me to get very close to God and to make the world more peaceful. (At times, I also wonder if I can walk on water or float but am less certain about this.)
Annabelle	Single	Unemployed	SzA	AMHT	Past	I have been chosen by God to be the only one he speaks to because I am special, his favourite, and his daughter. People will build temples dedicated to me.
Sophie	Engaged	Employed‐PT	BD	EIP	Past	I am God. I have the power to walk on water and bless people. I will save the world.
Stephen	Single	Unemployed	S	AMHT	Current (regarding past abilities)	I have special powers (to read minds, levitate objects, and travel through time). I am God. I have slept with billions of women and fathered children by them. I created and starred in the Avengers (who are real).
Max	Single		SzA	AMHT	Past	I am on an MI7 training programme; I am in the SAS; I am 007.
Sonja	Married	Unemployed	BD	AMHT	Current	I have special abilities to access and transfer information via telepathy (including with the spiritual realm). I can read others’ minds extremely quickly (much more quickly than others.)
Jessica	Divorced	Unemployed	BD	AMHT	Current (regarding past abilities)	I am able to do telepathy. I have special knowledge (the ability to predict the future) and abilities (knowing codes to enter and exit locked buildings). I am on a special mission of great importance.
Fred	Single	Unemployed	S	AMHT	Current	I am a messiah, God‐like figure for the world. I have superior consciousness compared to other people.
Sarah	Divorced	Unemployed	S	Inpatient	Current	I receive visions from God which allow me to predict the future. God has given me this ability because I am the Holy Spirit and his representative on earth. God kills those who harm me because I am special.
Brian	Single	Unemployed	Non‐organic psychotic disorder	EIP	Current	I am the reincarnation of Albert Einstein and have advanced mathematical abilities.
Polly	Single	Unemployed	S	AMHT	Current	I have been chosen by God to have a special role in saving the world. I will do this by marrying a person identified to me by God (either a current friend, or Jesus himself).
Mildred	Married	Employed‐FT	BD	AMHT	Past	I have special powers to predict the future; I have been chosen by God to save the world from evil forces because I am special.

Note

AMHT = Adult Mental Health Team; BD = bipolar affective disorder; EIP = early intervention for psychosis service; FT = full‐time; PT = part‐time; S = schizophrenia; SzA = schizoaffective disorder.

^a^ One participant was open to AMHT at the time of interview but had been recruited during his admission to hospital, and the interview took place 1 day after discharge.

### Procedure

#### Interview guide evolution

Consensus meetings and pilot interviews (conducted with those who had personal experience of grandiose delusions) facilitated the development of a preliminary interview guide. Decisions made at this stage included starting the interview with an open unstructured question inviting participants to tell their story about their experience of the identified belief. This ensured that the participant could talk about the issues most important to them. It was also decided to have two versions of the interview questions to ensure that experiences related to both past and current beliefs could be discussed sensitively (e.g., asking ‘How did you come to **believe** you were God?’ or ‘How did you come to **realise** that you were God?’). Emergent themes were incorporated into the interview guide as they arose. For example, after the first five interviews, it became apparent that the experience of grandiose beliefs was not synonymous with feelings of superiority or arrogance and therefore an additional question was added to elicit additional information about this (Question: ‘I’m interested in how this experience impacts on your view of yourself in relation to others. Do you see yourself as different or the same as others?’ If difference was suggested, optional further probes included: ‘How are you different? Is this in a good way or a negative way? Do you see yourself as better or worse than others? Or superior or inferior to others?’).

#### Interview process

Semi‐structured, in‐depth, audio‐recorded interviews were conducted by the lead author in accordance with relevant guidelines (Byrne, 2011; Yeo *et al.*, 2014). They were open‐ended (59–187 min). After the initial open question, subsequent focused questions facilitated discussion about belief onset, possible maintenance factors, impact on the individual, and experiences of mental health services. Follow‐up questions and probes were used as appropriate. Interviews were transcribed, anonymized, checked for accuracy, and offered to participants for review (Bazeley, 2013; Poland, 1995).

Almost all participants were unknown to the interviewer (a clinical psychologist in the Trust) before the study. One participant had completed a course of therapy with the interviewer 2 years prior to the present study. Where possible, steps were taken to minimize potential power imbalances between the interviewer and the participants (Gilburt, Rose, & Slade, 2008; Hoffmann, 2007). These included the interviewer emphasizing their viewpoint that the participant’s perspective was paramount and that our intention was to learn from them. Care was also taken to schedule the interview at a time and location of the participant’s choice and to remind participants that they could choose not to answer questions. It was also emphasized that information given by the participant in the interview would only be shared with care teams in the presence of significant risk.

### Method of analysis

Transcribed data were read and re‐read to ensure familiarity with the data. All interviews were coded by the lead author, however in line with recommendations (Barbour, 2001), multiple coding for a number of interviews, team reviews of the coding framework, and regular team consultation, including where uncertainty arose during coding, aimed to increase reliability.

Two early transcripts were considered in their entirety by two members of the research team (LI and LG) who each independently recorded ideas for possible codes before discussing. Suggested codes were further discussed with a third team member (DF) who had also reviewed these early transcripts. A preliminary coding framework which therefore incorporated multiple perspectives on the data was subsequently agreed. This framework largely corresponded to overarching topics on the interview guide but evolved in line with emerging ideas.

Details regarding each code (including the specific data and coder it had originated from, and whether it was an *a priori* or ‘*in vivo*’ code) were recorded, using memos in Nvivo, to form a codebook. The coding framework was regularly reviewed by the research team and adjusted accordingly. For example, after coding of the first five transcripts, the coding framework for the potential maintenance mechanisms of grandiose beliefs was very ‘fine‐grained’, with 62 different codes. Team discussion regarding the relative costs and benefits of broad (‘lumping’) versus fine‐grained (‘splitting’) coding (Guest, MacQueen, & Namey, 2012b; Weller & Romney, 1988) yielded a refined framework (30 codes), where codes that turned out to be similar were merged together. For example, the code ‘positive impact of the grandiose belief on beliefs about the self’ initially had 11 associated sub‐codes describing different sub‐categories (e.g., ‘it makes me normal’, ‘I will have a better job than before’). In the revised framework, the sub‐codes were dropped, and their associated data subsumed into the broader code. Details of the original sub‐codes were recorded in the codebook, however, so that ideas were not lost and could be considered during analysis. Other coding reviews found that the codes ‘rang true’ with team members’ own experiences of grandiose beliefs, who felt key information was being captured helpfully within the framework.

In addition to the first two interviews being double‐coded (as described earlier), coding of an early interview was reviewed in its entirety by an additional coder (BS) to increase reliability. This yielded additional codes such as ‘behavioural enaction’ (capturing behaviours resulting from the grandiose belief) that were incorporated into the coding framework. An iterative approach was adopted in the coding phase. As the coding framework evolved, earlier interviews were reviewed to ensure information relevant to emerging codes was captured. For example, the ‘behavioural enaction’ code was added after the first five interviews had been coded and therefore these interviews were reviewed again to ensure that pertinent data from these transcripts was captured. After all interviews were completed, ‘coding checks’ of each transcript were conducted. This involved the primary coder reviewing their initial codes with ‘fresh eyes’ after a period of time which has been recommended as a strategy to mitigate against ‘distorting effects immersion in the data can cause’ (Guest *et al.*, 2012a).

Interviews were explored using inductive and deductive thematic analysis (Braun & Clarke, 2006), and drawing on grounded theory whereby the detailed investigation of initially unstructured narratives was compared to the research question under investigation. This offered a high degree of flexibility and fidelity to the data. As indicated, this approach generated initial codes, which were constantly compared and modified as new interviews were added and analysed. This allowed for the initial formation of conceptual themes which were constantly re‐examined by the addition of new data in a dialectical process (Hutchison, Johnston, & Breckon, 2010). NVivo version 12 was used to support the coding, organization, and analysis of data.

## Results

### Harm

*Interviewer:* ‘I wondered whether you’d be able to tell your story […] of your experience of being Jesus?’

*Kit:* ‘Well, first off, it’s ten years of being sad’.

Harmful or potentially harmful situations were identified by all participants and had arisen in multiple life domains as a direct consequence of their grandiose beliefs. Trying to fly or walk on water (physical harm), going home with strangers they believed to be God (sexual harm), being rejected or ridiculed by others for their beliefs or associated behaviours (social harm), dropping out of university because of preoccupation with experiences (occupational harm), and feeling depressed, frightened, angry, under pressure, and suicidal (emotional harm) were all described (Box 1 provides additional quotes).

Box 1 **Further examples of harm across domains**

**Physical harm**

*Sophie:* “In some cases I wouldn’t think through where I tried [walking on water]. So maybe it will incidentally be shallow […] but also in deeper places, and […] places where getting out might have been challenging”; “It could’ve gone very very wrong if things had been slightly different […]. I could’ve got seriously hurt.”

*Sophie:* “Trying to fly off various heighted objects”; “[I] stepped off things and expected to fly.” Interviewer: “What’s the highest thing you’ve stepped off?” Sophie: [deep exhale, 10s pause]“I can’t entirely remember. And I don’t want to remember if that makes sense.”

*Max (describing an altercation at a nightclub whilst believing he had secret services training and protection from ‘other’ officers):* “Normally I would’ve just left it but […] because I felt that I was in some sort of training scheme, some organisation I felt a lot more confident so that added to the conflict. […] I felt that people were looking out for me.”

*Jessica:* “I was on a mission […] I walked across fields, I took my shoes off and put them as markers […]. I ended up walking, I’ve never seen it before but there was a caravan and I knocked on the caravan and this man was startled, as you would be at 11, 10 at night. But he wouldn’t let me in. And it was absolutely chucking it down, and maybe I wouldn’t, but bearing in mind I’ve got not shoes and socks.”

*Brian (talking about being Einstein):* “I needed to get to the highest point, so I could see, like, the horizon line […]. And that’s when they sectioned me because they thought I was going to commit suicide because I was over like loads of electric wires […]. I was on the lamp post on the bridge, sitting on top of it. […] I just wanted to see the horizon line. I was literally just obsessed with space and that.”

**Sexual harm**

*Kit:* “I have met with my Father [God] twice in human form. […] first one was Arthur* and Arthur* was a bit confusing […]. What he does he tries to give me like life lessons […] but then he also wanked off to gay porn when I was in the room and I felt a bit violated.”

Polly’s description of sexual harm is presented in the text. The example given however was not an isolated incident and Polly described several similar occurrences including one when she ended up spending the night on the streets with a homeless man who she described as being high on narcotics.

**Social harm**

*Stephen:* “I was talking to her, I was gonna offer her a drink, and this other girl pulled her away and said ‘ I just thought I would pull you away from that situation’ […]” Interviewer: “Why do you think she did that?” Stephen: “I don’t know. It’s just what people think I am isn’t it […]. People think I’m a weirdo. Some people think I’m not right in the head.”

*Mildred (describing a previous boyfriend ending their relationship when she believed she was in a battle of good vs. evil with one of his relatives):* “He was just like, ‘I can't… I just can't do this anymore.’” Interviewer: "And what impact did that have on you at the time?" Mildred: "Erm…. my world fell to pieces."

**Emotional harm**

*Fred (describing feeling different to others as a Messiah):* “In my 30s I wanted to die; I wanted to commit suicide […] For anyone in that position I thought it would be ordinary to commit suicide, it was just hopeless”; “I was certainly depressed for a long time, and I came to this momentous decision, ‘oh, to hell with it all, I’m not playing this game of being a human being anymore’.”

*Jessica:* “There were fireworks going off but to me they weren’t fireworks, they were gunshot rings and I remember, although I was scared and that, I was on a mission, I had to do it.”

*Bob:* “The messiah is completely devoid off all sin […]. So I would not allow myself […] to feel any greed, […], any sort of desire, without feeling guilt for it, without feeling self‐hatred.”

*pseudonym.

Harm was sometimes the direct consequence of the participant’s behaviour (Jessica: ‘I drove faster than I normally would’) but frequently the risk came from others. Some, especially the male participants, knowingly entered dangerous situations feeling themselves to be invulnerable (Max described confidence during an altercation at a nightclub because ‘I felt that people were looking out for me’). Others demonstrated a lack of awareness of the risks posed by others:

*Polly:* ‘This elderly gentleman came up to me […]. I thought “you’re God”. I went to his house […]. We had some kisses and cuddles and I said “can we be married?”. He said “no”. […] “we can be partners” and from that I thought he meant not literally romantic partners but business partners; partners in the process of saving people’.

Often the participant was adversely affected but there were examples of significant harm to others, with evidence of family, friends, and strangers experiencing distress, neglect, embarrassment, or fear:

*Max:* ‘I saw two guys […] and said “stop, I want to speak to you”. […] [They] started walking away. I don’t know if they were doing something dodg‐, but then I opened my jacket and went like [motions reaching inside inner jacket pocket]. They started running […]. I said “stop armed police!” or something and they just ran off’.

*Sarah:* ‘I was going to heaven, […] spending time with God […]. Always in visions [dreams]. My days would be perfectly normal, but my nights would be just magical. And this is where we get to my daughter because… I just wanted to go to bed. She was a teenager and wanted to be out with her friends and I would just ignore her. Go to sleep and leave her. I didn't even know what time she was coming in. […] It did impact our relationship. […] I would go to bed early […]. say seven o'clock, […] because that was more exciting than my daily life and I didn't realise that I neglected her’.

Harms were evident both when the belief was present but also afterwards. Participants recalled feeling embarrassment or a sense of loss once the belief receded (Max: ‘you slip into quite a deep depression after you realise […] it’s not like you go from a feeling of being really important back to where you were before, you go from really important to really unimportant’.). Others described encountering practical difficulties, such as Sophie who described the impact of taking time off work due to a hospital admission that was directly related to her belief:

*Sophie:* ‘It’s practically hugely damaging. Seven weeks off work – big problem. After seven weeks I missed out on the chance to do my [job specific] certificate […]. I was just getting the management to agree to support me, getting my mentor, I’d done all the work, they just needed to sign it off, and then I was in [hospital city] for seven weeks. […] Actually no […] there’s like a three, four week gap, then another five weeks where I’m not working, then two or three weeks of day hospital afterwards. Suddenly it’s been that long, you go back [to work] but not all the same staff are there, it was a different manager. I was no longer so regular and valued that they wanted to do it, and I was still impaired. I don’t know why I was still impaired, I don’t know why everything’s harder but after that everything was so much harder’.

Harm was not solely caused by the belief *per se*, but sometimes due to the degree of preoccupation with it (Mandy described accidentally scalding herself whilst caught up thinking about the belief) or by others’ responses:

*Mandy:* ‘My brother’s partner said “can Mandy come up?” and‐ …I was very upset once because my cousin said “No. I can’t cope with what she’s saying [about being the Goddess], it’s stressful for me […]”. So I couldn’t sometimes go up’.

Disbelief by others was prominent and experienced negatively by most participants, especially those currently hospitalized:

*Kit:* ‘I was going to kill myself on New Year’s Eve […] It was linked to breaking up with my girlfriend and ten years of just people ignoring me [Jesus] [….], I even went to the Evensong, you know, in a church, stood next to everyone, they were all singing to Jesus, and no one fucking talked to me. No one really does want me [Jesus] because, you know, it lasts a lot longer if I’m just dead and people just don’t know’.

### Maintenance mechanisms

Six potential psychological maintenance factors were identified. Box 2 provides further illustrative quotes.

Box 2 **Illustrations of possible maintenance mechanisms and service‐related experiences**

**Meaning‐making experiences**

**Helping others and hope for the future:** *Mandy:* “I was telling me parents I could help ‘em […]. They’re suffering now but it will come out alright.”; “[God] says ‘hang on in there’ […] I know you suffer sometimes but suffering's for a reason, and you will come out of it.’"

**Power and achieving potential:** *Max:* “[It made] me feel strong and powerful and sort of able to do anything. The sort of feeling you get, it makes you feel like you become the person you’ve always wanted to be or better.”

**Being useful and helping society:** *Mark:* “I feel I am useful to society.”

**Social meaning:** *Stephen:* “I just feel part of a team”; *Mandy*: “I’m gonna have children in the new life.”

**Anomalous experiences**

**Anomalous experiences powerful and intense, increasing sense of their significance:** *Kit:* “The actual powerful voice of God spoke to me and said ‘Do it right this time.’ I fell into a bush […] like it came out of kind of sunlight clouds which was on the righthand side of me. And it was so powerful I fell over.”

**Anomalous experiences being sought due to their meaning in the context of the grandiose belief:** *Kit:* “God reveals himself to people in dreams. So my dreams have always been the most interesting thing that I spend a lot of time asleep dreaming and I force myself to sleep to dream because God shows himself in that way.**”**

**Mania**

**Mania preceding development of grandiose belief:** *Mildred:* “For that particular episode,[…] I know exactly where the trigger came from. My mood had started to go up and I was reading these books[…] I think I managed to get through all ten within about two weeks. […] I was sleeping less than I normally do, but […] I wasn't worried about it. […] I think my mood went up before the sleep reduced”.

**The grandiose belief changing when the mania recedes:** *Mildred:* “I think I just came out of my episode, basically. I think natural… I go up and down. I literally naturally came out of the other side and my focus just ever so slightly shifted”.

**Fantasy elaboration**

**Thinking about the belief (in imagery form) feels good:** *Mandy:* “Well it can feel good, yeah, looking like that [giggles]. I could see err‐, see myself, err the eyes they are not just err… they’re like that! [gesturing large eyes]”.

**Reasoning biases**

**Confirmation bias:** *Bob:* “I had the ideas…. It became a reciprocating system in that I would then feel this reinforcement with this information stream. […] As my perception would change, the information stream would change. In much the same way, if you are ice skating and you start looking one way you will start drifting that way.”

“There was a pathway which I followed of my own logic, which was potentially fallible […]. But I didn’t take the time to try to fail myself […] because failing myself would mean the past few months I had done had gone to waste and I'd destroyed myself and the whole post negative implications which I did not want to face.”

**Negative social information being misinterpreted positively:** *Polly:* “An elderly gentleman […] walked past me […]. I thought ‘he looks like God’. […] I said, ‘Hello, Daddy’, and he said something like ‘what do you want?’ [hostile tone]. I said, ‘What can I do to please you?’ […] He said, ‘Nothing.’ I said, “What can I do for you?” And he said, ‘nothing!’” Interviewer: “What was that like?” Polly: “Well, it was nice to meet him.” Interviewer: “When he said, ‘there's nothing’, what did you take from that?” Polly: “That Jesus has done it all, we don't have to”.

**Advice/feedback from others rejected:** *Bob:* “Anyone who tried to come and sort of say ‘No, sorry your reality's false**,** you are completely psychotic’ had no effect on me, except just to sort of aggravate… umm… and to push me further away.”

**Immersive behaviours**

*Sophie:* “I was completely convinced I was God. I needed to go out and bless via libraries. Why libraries I don’t know, but I was convinced that libraries were an effective way to bless and was just going around… yeah.”

**Service‐experience**

**Positive techniques used to help manage the grandiose delusion:** *Participant:* “my CPN was amazing […] thinking about thinking patterns and cycles of behaviour […] ways to challenge it, looking a bit at the evidence and like noticing reinforcing patterns.”

*Participant:* “If you want to approach this problem for grandiosity […] you need to approach many other things in life. You may find that […] you still have that grandiosity at the end of it, but it wouldn’t be a problem. […] It’s more about making a person a more well‐rounded individual. The problem isn’t grandiosity, the problem is how they view themselves, how they interact with the world.”

#### Meaning‐making

All narratives emphasized that grandiose beliefs were ‘meaning‐making’ experiences. Participants reported the beliefs as highly significant and they appeared to provide a sense of purpose, belonging, or self‐identity, or make sense of unusual or difficult events.

The types of meaning inherent in the belief differed between participants. Power and self‐efficacy, helping others, and making a valuable contribution to society were common themes. Social meanings were also prevalent and participants described that they were (or would be) ‘part of a team’, respected by others, or involved in intimate relationships with the promise of comfort, protection, marriage, sex, or children.

Frequently grandiose beliefs occurred in the face of negative circumstances and, as such, appeared to be protective. Accounts of the belief providing respite from paranoia, low mood, self‐loathing, and rejection, and as a means to make sense of suffering, achieve retribution for past wrongs, and retain hope for a better future were all described.

*Bob:* ‘I hated who I was.’; ‘I tried to seek some sort of route to escape this depression, which was to again fall into this fantasy world in which I would try to elevate myself, and you can elevate yourself as far as you want in your own fantasy, you can be the next messiah […].’

Such a ‘meaning‐making’ function could lead to belief persistence:

*Bob:* ‘[I] wanted the fantasy to persist, […] I wanted to be Messiah, I wanted to be important’; ‘I wasn’t looking for information against it because I didn’t want it to be false’.

The inherent meaning was not typically synonymous with feeling highly superior, arrogant, or overly entitled. When superiority was evident, it was often not totally unwarranted (e.g., the participant having above average intelligence), or it was accompanied by humility or uncertainty:

*Fred:* ‘I feel superior to other people, definitely, yes. I don't go around saying that, […] but that's how I feel inside’.

*Interviewer:* ‘Having these abilities, do you see yourself as different to others in some way, or the same, or…?’ Jessica: ‘No, no. No, not at all.’; Interviewer: ‘So when you felt you were on a mission, […] in that moment have you felt better or worse than others, or superior or inferior to others? […]’ Jessica: ‘No. Probably the same’.

*Interviewer:* ‘Does [being the Goddess] make you feel better than other people?’ Mandy: ‘No no. ‘Cause we all come from‐. In fact they‐, everybody comes from me in the first place, flesh was took away from me, but umm…no no. I would be over [them] in a way, but no, all people are people. They should all be treated the same’.

*Polly:* ‘I **know** I’m not better than anybody else…, but it does make me feel special’. […]. Interviewer: ‘Do you feel superior to others?’ Polly: ‘I do but that’s rubbish, I shouldn’t feel like that’.

#### Anomalous experiences

Anomalous experiences (AEs) were described by all but one participant. Most common were auditory hallucinations (reported by eight participants) and a felt sense of salience (reported by six participants) but other hallucinations (somatic, olfactory and visual), dissociative experiences (out of body experiences and déjà vu), and vivid dreams were also evident.

Anomalous experiences were implicated in belief maintenance in several ways. First, the content of the AE could cause or confirm the belief. Mandy described realizing she was the Goddess when ‘He [God] was in my head and telling me.’; ‘A voice was telling me’. Similarly, Sophie described a referential belief (‘the sunset told me stuff, it had meaning’), underpinned by a felt sense of salience, which fed into her belief about having special abilities. The presence of an AE was often described as the defining moment at which the person ‘knew’ their belief was true, and some indicated that the belief receded when the AEs did.

Anomalous experiences were described as powerful and intense, making them potentially more likely to be appraised as significant. For some, the grandiose belief was the most plausible explanation for AEs that felt strange and profound:

*Fred:* ‘I had an immense shift of consciousness, rather like suddenly being able to see, whereas previously I couldn't see.’; ‘I felt that something momentous had happened […] I attributed it to being the second coming of Christ, because that was the only framework that I had to put it in’.

A reciprocal relationship was also evident with some participants deliberately seeking out AEs because they were seen as important or pleasurable in the context of the grandiose belief (Kit, Sarah).

#### Mania

Where grandiose beliefs co‐occurred with mania, interactions were sometimes apparent. Max said ‘[The grandiose beliefs] have always been after elevated mood.’, and ‘It’s a really good feeling, feeling that you’re in the SAS’ suggesting a bi‐directional interaction with elevated mood. Brian described racing thoughts (‘the numbers started coming really fast’) contributing to his realization that he was Einstein reincarnated, and several participants described sleep disturbance preceding or accompanying their grandiose beliefs.

Mania was not a necessary condition for the maintenance of grandiose delusions, however, and several participants (including those with affective diagnoses) presented at interview with current grandiose beliefs in the absence of elevated mood/mania. Mildred noted that of two occasions when she believed she was chosen by God to battle evil, one was clearly preceded by ‘mania’ (elevated mood, poor sleep, increased energy) and resolved when she ‘came out of my episode’ but that mania was not present on the other occasion:

‘I don't know what triggered that, only that my Dad had left, […] my Mum had a nervous breakdown, so I was left in charge of my two younger sisters’; ‘I suppose it was obviously very heightened emotionally, so it must have… it can only have come from that, but I don't remember having a particularly high mood’; ‘[the other experience] was different. There was a lot of energy behind that that there wasn't with this’.

#### Fantasy elaboration

Participants described thinking about their grandiose beliefs ‘all the time’ (Mark; Polly), that ‘it took over my whole life’ (Brian), and that it was only possible to stop thinking about them when significantly distracted (e.g., helping someone in trouble (Bob) or starting a new company (Mildred)). Such thoughts were not always verbal; compelling images were also present:

*Mandy (describing an image of being the Goddess):* ‘I’m blonde hair, big brown eyes, and they’re massive […]. I felt these huge eyes and long blonde hair, and then a figure’.

Whilst we anticipated that repetitive thinking would occur because it was pleasurable, the wider meaning, which typically went beyond simple hedonic pleasure, also drove repetitive thinking:

*Mark:* ‘It fills my time. I'm always busy […]. In the past without doing that I'd be just feeling bored, sitting in my flat, listening to the radio, watching TV, sitting on my computer, bored, drinking alcohol. […]. But with this situation I am busy thinking all the time’.

#### Reasoning biases

Participants’ descriptions were consistent with a range of biases being present, most commonly confirmation bias:

*Interviewer:* ‘If someone had said “we don’t think that is happening” […] how would you have reacted?’ *Mildred*: ‘Well…. That [would be] just another sign than I’m on the right path. That’s a test’.

Jumping to conclusions (an absence of data‐gathering) also occurred:

*Max:* ‘I spent a lot of time thinking about it, not that much time like researching about it. Just thinking, thinking about it and feeling I would get the right answers myself without actually looking it up’.

Negative social information was misinterpreted as positive, and there were descriptions of discrediting advice or feedback:

*Stephen:* ‘People just kept staring at me wherever I went […]’. *Interviewer:* ‘What did you conclude from that?’ *Stephen:* ‘That I was something powerful’.

*Interviewer:* ‘When you’re in that mode of being God, how do you respond to advice or feedback from others?’ *Sophie:* ‘Completely dismiss and ignore it’.

Although reasoning biases were frequently evident, there were counter‐examples including altering belief conviction with disconfirmatory evidence:

*Max:* ‘I was convinced that I was in the SAS […], I thought the police were gonna raid the place and get me out. And obviously that didn’t happen, so I think when I came out I felt a bit less convinced’.

#### Immersion behaviours

Participants described behaviours where they immersed themselves in a world consistent with the delusion. This included acting according to their perceived role or identity (Sophie: ‘I was God. I needed to go out blessing’; Max: ‘I was in the SAS […] I was sort of patrolling the town’), or withdrawing and becoming engrossed in information that fitted with their belief (Bob: ‘I shut myself off from the world […] I was sort of in my brain with videos online, articles, and on the internet there’s no filter, you can literally get anything. I was […] trying to get in touch with what I thought reality was’).

Participants described engaging in these ‘immersion behaviours’ for several reasons. Some wanted information to understand how to achieve their ‘mission’ or evidence to prove to themselves or others that their belief was true. Others acted because it felt good or important. Sophie described trying to walk on water with differing rationales. When uncertain if she was a demi‐God, she ‘did some experiments to test [it] out’, but when she ‘knew’ she could do it she acted because ‘it could be fun’.

### Experience of service‐use and help‐seeking

*Participant:* ‘Nobody talked to me. I wanted to talk to them […] I was alone and isolated’.

Participants unanimously reported difficulty talking to mental health services about their experience of grandiose delusions, despite the majority thinking that it might be helpful. Experiences were reported as hard to articulate (Fred: ‘it’s very hard to […] know what to say to describe it’) or secrecy was inherent in the belief (Max: ‘I won’t speak to them about it, thinking it’s something that needs to be kept secret’). The lack of discussion was primarily attributed to staff‐ or service‐related factors. Staff not knowing how to talk about grandiose beliefs, speaking to family members rather than the participant, or simply not listening or understanding were described. Insufficient time in appointments or previous aversive experiences (e.g., compulsory admission, or feeling ‘browbeaten’, ‘ignored’, or ‘dismissed’) were further barriers to opening up:

*Participant:* ‘You tell care staff, the medical staff and then they say, “right, you have to go into hospital” and “we're taking your driving licence away”’.

Talking about the grandiose belief was considered important to enable risk monitoring, facilitate belief change, or offer support:

*Participant:* ‘Even if you can’t change my beliefs I really appreciate being listened to and talked to ‘cause it’s really upsetting […]. You can do that human support even if you can’t change the situation’.

In terms of what would be helpful, taking time to develop trust was repeatedly reiterated. Other recommendations included asking specifically about the experiences (without being pushy) and listening carefully to the participant’s perspective:

*Participant:* ‘If people don’t take the time to get to know, and don’t ask questions […] it’s a big problem. Because if I’m having these ideas I think it’s obvious. […] It’s quite unhelpful when people assume you’ll tell them stuff. […] So actually try to talk about it and interact with it, rather than just assuming you’ll tell people everything.’

Participants particularly appreciated staff who had ‘gone the extra mile’ (e.g., buying the participant a coffee or taking extra time to talk when distressed).

Few participants had been offered therapy for their grandiose beliefs. Unhelpful experiences of therapy more generally included too great a focus on the past or the participant feeling blamed (‘[it’s] your thought processes that were wrong, […] there’s something wrong in you’). However, descriptions of helpful therapy experiences suggested that looking at evidence for and against the belief, considering alternative explanations, and looking at aspects identified as possible maintenance cycles may be beneficial:

*Bob:* ‘[Good therapy would be] something that makes them feel good, […] makes them want to be in reality. Getting up every day, going for a morning run, having some good breakfast […] having projects to work on, having skills you learn. […] What’s your lovelife like?[…] You need to look at all aspects of the person’s life.’; ‘You’ve also got to have a sense of belonging […] a place within your society, a sense you have some worth’.

## Discussion

This is the first qualitative study focussed upon the experience of grandiose delusions. The patient accounts were extraordinarily rich, with most participants never having spoken in depth before about these experiences. Harm from grandiose delusions – across multiple domains – was evident for all the participants, and occurred as a direct consequence of the belief, from preoccupation, and from the responses of others. The limited literature on harm associated with grandiose delusions focuses almost exclusively on offending (van Dongen, Buck, & Van Marle, 2015; Ullrich, Keers, & Coid, 2014) but clearly a wider perspective is needed.

**Figure 2:**
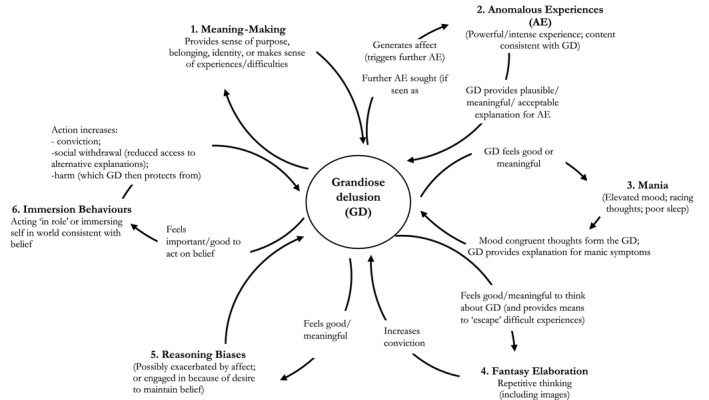
Hypothesized maintenance model of grandiose delusions. NB: Not all maintenance factors were evident in all participants. As such, we suggest that no maintenance factor is either necessary or sufficient for the persistence of grandiose delusions, and idiosyncratic combinations of factors will be relevant to different individuals.

A number of potential maintenance factors were identified (see Figure 2). Foremost, the beliefs provided a sense of purpose, belonging, or positive identity, often in difficult circumstances, creating a motivation for belief retention. Second, grandiose beliefs offered a plausible explanation for anomalous experiences, which, in some cases, resulted in these experiences being actively sought. Third, a mood‐elevating bi‐directional relationship between symptoms of mania and grandiosity appeared to occur for some patients. Fourth, positive rumination or ‘fantasy elaboration’ may act in a way akin to that of worry in persecutory delusions (Freeman *et al.*, 2015), whereby repetitively thinking (or having imagery) about the belief brings it to mind, elaborates details, and increases conviction. Fifth, reasoning biases were also prominent, consistent with evidence that they are heightened in grandiose delusions (Garety *et al.*, 2012). Negative social feedback appeared to be disregarded or interpreted in an overly positive manner, similar to findings in hypomania (Devlin, Zaki, Ong, & Gruber, 2015; Mansell & Lam, 2006). Finally, immersive behaviours reinforced the belief. Memories for self‐performed actions may be stronger compared to imagined actions (Engelkamp, 1989), so that ‘being in role’ may provide particularly accessible or compelling memories.

These findings from patient interviews were consistent with hypotheses considered by other researchers who have suggested that grandiose beliefs may compensate for negative self‐beliefs (Beck & Rector, 2005; Ben‐Zeev *et al.*, 2011; Knowles *et al.*, 2011; Smith, Freeman, & Kuipers, 2005), and be associated with anomalous experiences (Bortolon *et al.*, 2019; Knowles *et al.*, 2011), reasoning biases (Garety *et al.*, 2012; Knowles *et al.*, 2011), and repetitive, imagery‐based thinking (Knowles *et al.*, 2011). Further research empirically testing the hypothesized maintenance model, and determining the extent to which specific maintenance factors are unique to delusion subtypes, is clearly required.

The qualitative nature of our investigation enabled a hypothesized maintenance model for grandiose delusions to be generated; however, there were some limitations. Obviously, our findings are not representative, and we did not include those with subclinical grandiosity or older adults, nor gain viewpoints from other key groups (e.g., family members or mental health professionals). The sample were predominantly White British and although this represents the demographic structure of the capture area of the NHS Trust in which the study took place, the transferability of the findings may therefore be limited. There may be other potential maintenance factors that we did not identify within this study. Additionally, although we took multiple steps to minimize the potential for bias as much as possible, our own experiences (as clinical psychologists, qualitative methodologists, and those with personal experience of grandiose delusions), and the fact that only a small subgroup of interviews were coded in full by multiple coders, mean that data were viewed through a particular lens. Further research investigating different populations and viewpoints would be of value.

Despite these limitations, such models have the potential to drive clinical interventions in the future, and there were several key implications from the participant interviews that should be considered. The level of harm evident highlights the need for a targeted treatment specifically for grandiose delusions. Patient recognition of some forms of harm indicates a possible route for engagement, and participants were largely positive about the possibility of receiving psychological therapy. Any decision to intervene, however, should only be made after careful consideration of the meaning and associated benefits of the belief. Trying to alter the belief without first compensating for the benefit or function of the belief is likely to prove both difficult and potentially iatrogenic. Direct belief change may not always be the most advantageous option. If harm is limited to negative responses from others, addressing behavioural responses to the grandiose belief (e.g., discerning who can be talked to about the experiences) and taking steps to address stigma more broadly might be more appropriate.

Notably, grandiosity was not synonymous with high levels of superiority, arrogance, or entitlement. This is significant because ‘grandiose’ is often used as a derogatory term to indicate such traits. It may be that grandiose beliefs enhance self‐esteem, but do not necessarily cause it to become excessively exaggerated. Alternatively, as suggested by one participant, such traits, when apparent, may be more closely connected to mania. Since actually having exceptional abilities or identity is not synonymous with viewing oneself as being inherently better than others, then superiority should not be assumed to occur in the context of grandiose delusions. Consequently, we suggest that if this finding is replicated in future studies, grandiose delusions should be better termed: ‘delusions of exceptionality’. This may be a more accurate reflection of the experience and, as such, a better way to think about administering care.

## Conflicts of interest

All authors declare no conflict of interest.

## Data Availability

Given the confidential nature of the data, it would not be ethically appropriate to share the entire data set (i.e., whole transcripts). Selected quotes to support claims made in the paper however are available on request to the first author.

## References

[papt12260-bib-0001] Appelbaum, P. S. , Robbins, P. C. , & Roth, L. H. (1999). Dimensional approach to delusions: Comparison across types and diagnoses. American Journal of Psychiatry, 156, 1938–1943. 10.1176/ajp.156.12.1938 10588408

[papt12260-bib-0002] Barbour, R. S. (2001). Checklists for improving rigour in qualitative research: A case of the tail wagging the dog? BMJ (Clinical Research Ed.), 322, 1115–1117. 10.1136/bmj.322.7294.1115 PMC1120242 11337448

[papt12260-bib-0003] Bazeley, P. (2013). Qualitative data analysis: Practical strategies. Evaluation and program planning (Vol. 1). London: Sage.

[papt12260-bib-0004] Beck, A. T. , & Rector, N. A. (2005). Cognitive approaches to schizophrenia: Theory and therapy. Annual Review of Clinical Psychology, 1, 577–606. 10.1146/annurev.clinpsy.1.102803.144205 17716100

[papt12260-bib-0005] Ben‐Zeev, D. , Morris, S. , Swendsen, J. , & Granholm, E. (2011). Predicting the occurrence, conviction, distress, and disruption of different delusional experiences in the daily life of people with schizophrenia. Schizophrenia Bulletin, 38, 826–837. 10.1093/schbul/sbq167 21248277 PMC3406533

[papt12260-bib-0006] Bleuler, E. (1950). Dementia praecox or the group of schizophrenias. Oxford, UK: International Universities Press.

[papt12260-bib-0007] Bortolon, C. , Yazbek, H. , Norton, J. , Capdevielle, D. , & Raffard, S. (2019). The contribution of optimism and hallucinations to grandiose delusions in individuals with schizophrenia. Schizophrenia Research, 210, 203–206. 10.1016/j.schres.2018.12.037 30639163

[papt12260-bib-0008] Braun, V. , & Clarke, V. (2006). Using thematic analysis in psychology. Qualitative Research in Psychology, 3(2), 77–101. 10.1191/1478088706qp063oa

[papt12260-bib-0009] Byrne, B. (2011). Qualitative interviewing. In C. Seale (Ed.), Researching society and culture (pp. 207–226). London: Sage.

[papt12260-bib-0010] Devlin, H. C. , Zaki, J. , Ong, D. C. , & Gruber, J. (2015). Tracking the emotional highs but missing the lows: Hypomania risk is associated with positively biased empathic inference. Cognitive Therapy and Research, 40(1), 72–79. 10.1007/s10608-015-9720-6

[papt12260-bib-0011] Engelkamp, J. (1989). Memory for actions. Hove, UK: Psychology Press.

[papt12260-bib-0012] Freeman, D. (2016). Persecutory delusions: A cognitive perspective on understanding and treatment. The Lancet Psychiatry, 3, 685–692. 10.1016/S2215-0366(16)00066-3 27371990

[papt12260-bib-0013] Freeman, D. , Dunn, G. , Startup, H. , Pugh, K. , Cordwell, J. , Mander, H. , … Kingdon, D. (2015). Effects of cognitive behaviour therapy for worry on persecutory delusions in patients with psychosis (WIT): A parallel, single‐blind, randomised controlled trial with a mediation analysis. The Lancet Psychiatry, 2, 305–313. 10.1016/S2215-0366(15)00039-5 26360083 PMC4698664

[papt12260-bib-0014] Freud, S. (1911). Psycho‐analytic notes on an autobiographical account of a case of paranoia (dementia paranoides). Worcestershire, UK: Read Books Ltd.

[papt12260-bib-0015] Fusch, P. I. , & Ness, L. R. (2015). Are we there yet? Data saturation in qualitative research. The Qualitative Report, 20, 1408–1416. Retrieved from http://www.nova.edu/ssss/QR/QR20/9/fusch1.pdf

[papt12260-bib-0016] Garety, P. A. , Gittins, M. , Jolley, S. , Bebbington, P. E. , Dunn, G. , Kuipers, E. , … Freeman, D. (2012). Differences in cognitive and emotional processes between persecutory and grandiose delusions. Schizophrenia Bulletin, 39, 629–639. 10.1093/schbul/sbs059 22499781 PMC3627767

[papt12260-bib-0017] Gilburt, H. , Rose, D. , & Slade, M. (2008). The importance of relationships in mental health care: A qualitative study of service users’ experiences of psychiatric hospital admission in the UK. BMC Health Services Research, 8(1), 92. 10.1186/1472-6963-8-92 PMC2386459 18439254

[papt12260-bib-0018] Goodwin, F. K. , & Jamison, K. R. (2007). Manic‐depressive illness: Bipolar disorders and recurrent depression. Oxford, UK: Oxford University Press.

[papt12260-bib-0019] Guest, G. , MacQueen, K. M. , & Namey, E. E. (2012a). Applied thematic analysis( pp. 79–106). Riverside County, CA: Sage.

[papt12260-bib-0020] Guest, G. , MacQueen, K. , & Namey, E. (2012b). Applied thematic analysis (pp. 73–75). Riverside County, CA: Sage.

[papt12260-bib-0021] Hoffmann, E. A. (2007). Open‐ended interviews, power, and emotional labor. Journal of Contemporary Ethnography, 36(3), 318–346. 10.1177/0891241606293134

[papt12260-bib-0022] Hutchison, A. J. , Johnston, L. H. , & Breckon, J. D. (2010). Using QSR‐NVivo to facilitate the development of a grounded theory project: An account of a worked example. International Journal of Social Research Methodology, 13, 283–302. 10.1080/13645570902996301

[papt12260-bib-0023] Knowles, R. , McCarthy‐Jones, S. , & Rowse, G. (2011). Grandiose delusions: A review and theoretical integration of cognitive and affective perspectives. Clinical Psychology Review, 31, 684–696. 10.1016/j.cpr.2011.02.009 21482326

[papt12260-bib-0024] Leff, J. P. , Fischer, M. , & Bertelsen, A. (1976). A cross‐national epidemiological study of mania. The British Journal of Psychiatry, 129, 428–442. 10.1192/bjp.129.5.428 990656

[papt12260-bib-0025] Mansell, W. , & Lam, D. (2006). “I Won’t Do What You Tell Me!”: Elevated mood and the assessment of advice‐taking in euthymic bipolar I disorder. Behaviour Research and Therapy, 44, 1787–1801. 10.1016/j.brat.2006.01.002 16487480

[papt12260-bib-0026] Mulder, C. L. , Koopmans, G. T. , & Hengeveld, M. W. (2005). Lack of motivation for treatment in emergency psychiatry patients. Social Psychiatry and Psychiatric Epidemiology, 40, 484–488. 10.1007/s00127-005-0913-2 16003598

[papt12260-bib-0027] Poland, B. D. (1995). Transcription quality as an aspect of rigor in qualitative research. Qualitative Inquiry, 1, 290–310. 10.1177/107780049500100302

[papt12260-bib-0028] Richie, J. , Lewis, J. , & Elam, G. (2014). Designing and selecting samples. In J. Ritchie , J. Lewis , C. McNaughton Nicholls , & R. Ormston (Eds.), Qualitative research practice: A guide for social science students and researchers (pp. 113–114). London, UK: Sage.

[papt12260-bib-0029] Ronald, A. , Sieradzka, D. , Cardno, A. G. , Haworth, C. M. A. , McGuire, P. , & Freeman, D. (2014). Characterization of psychotic experiences in adolescence using the specific psychotic experiences questionnaire: Findings from a study of 5000 16‐year‐old twins. Schizophrenia Bulletin, 40, 868–877. 10.1093/schbul/sbt106 24062593 PMC4059437

[papt12260-bib-0030] Smith, N. , Freeman, D. , & Kuipers, E. (2005). Grandiose delusions: An experimental investigation of the delusion as defense. The Journal of Nervous and Mental Disease, 193, 480–487. 10.1097/01.nmd.0000168235.60469.cc 15985843

[papt12260-bib-0031] Ullrich, S. , Keers, R. , & Coid, J. W. (2014). Delusions, anger, and serious violence: New findings from the macarthur violence risk assessment study. Schizophrenia Bulletin, 40, 1174–1181. 10.1093/schbul/sbt126 24048345 PMC4133660

[papt12260-bib-0032] van Dongen, J. , Buck, N. , & Van Marle, H. (2015). Unravelling offending in schizophrenia: Factors characterising subgroups of offenders. Criminal Behaviour and Mental Health, 25(2), 88–98. 10.1002/cbm.1910 24677735

[papt12260-bib-0033] Weller, S. , & Romney, A. (1988). Systematic Data Collection (pp. 23–29). Riverside County, CA: Sage.

[papt12260-bib-0034] Wing, J. K. , Babor, T. , Brugha, T. , Burke, J. , Cooper, J. E. , Giel, R. , … Sartorius, N. (1990). SCAN: Schedules for clinical assessment in neuropsychiatry. Archives of General Psychiatry, 47, 589. 10.1001/archpsyc.1990.01810180089012 2190539

[papt12260-bib-0035] Yeo, A. , Leagrd, R. , Keegan, J. , Ward, K. , McNaughton Nicholls, C. , & Lewis, J. (2014). In‐depth interviews. In J. Ritchie , J. Lewis , C. McNaughton Nicholls , & R. Ormston (Eds.), Qualitative research practice: A guide for social science students and researchers (pp. 177–210). London, UK: Sage.

[papt12260-bib-0036] Zavos, H. M. S. , Freeman, D. , Haworth, C. M. A. , McGuire, P. , Plomin, R. , Cardno, A. G. , & Ronald, A. (2014). Consistent etiology of severe, frequent psychotic experiences and milder, less frequent manifestations: A twin study of specific psychotic experiences in adolescence. JAMA Psychiatry, 71, 1049–1057. 10.1001/jamapsychiatry.2014.994 25075799 PMC4156464

